# Loss of MEF2D expression inhibits differentiation and contributes to oncogenesis in rhabdomyosarcoma cells

**DOI:** 10.1186/1476-4598-12-150

**Published:** 2013-11-27

**Authors:** Meiling Zhang, Jamie Truscott, Judith Davie

**Affiliations:** 1 Department of Biochemistry and Molecular Biology and Simmons Cancer Institute, Southern Illinois University School of Medicine, 229 Neckers Building, 1245 Lincoln Dr, Carbondale, IL 62901, USA; 2 Southern Illinois University School of Medicine, Springfield, IL 62794, USA

**Keywords:** Rhabdomyosarcoma, ERMS, ARMS, MEF2D, Myogenin and MyoD

## Abstract

**Background:**

Rhabdomyosarcoma (RMS) is a highly malignant pediatric cancer that is the most common form of soft tissue tumors in children. RMS cells have many features of skeletal muscle cells, yet do not differentiate. Thus, our studies have focused on the defects present in these cells that block myogenesis.

**Methods:**

Protein and RNA analysis identified the loss of MEF2D in RMS cells. MEF2D was expressed in RD and RH30 cells by transient transfection and selection of stable cell lines, respectively, to demonstrate the rescue of muscle differentiation observed. A combination of techniques such as proliferation assays, scratch assays and soft agar assays were used with RH30 cells expressing MEF2D to demonstrate the loss of oncogenic growth *in vitro* and xenograft assays were used to confirm the loss of tumor growth *in vivo*.

**Results:**

Here, we show that one member of the MEF2 family of proteins required for normal myogenesis, MEF2D, is largely absent in RMS cell lines representing both major subtypes of RMS as well as primary cells derived from an embryonal RMS model. We show that the down regulation of MEF2D is a major cause for the failure of RMS cells to differentiate. We find that MyoD and myogenin are bound with their dimerization partner, the E proteins, to the promoters of muscle specific genes in RMS cells. However, we cannot detect MEF2D binding at any promoter tested. We find that exogenous MEF2D expression can activate muscle specific luciferase constructs, up regulate p21 expression and increase muscle specific gene expression including the expression of myosin heavy chain, a marker for skeletal muscle differentiation. Restoring expression of MEF2D also inhibits proliferation, cell motility and anchorage independent growth *in vitro*. We have confirmed the inhibition of tumorigenicity by MEF2D in a tumor xenograft model, with a complete regression of tumor growth.

**Conclusions:**

Our data indicate that the oncogenic properties of RMS cells can be partially attributed to the loss of MEF2D expression and that restoration of MEF2D may represent a useful therapeutic strategy to decrease tumorigenicity.

## Background

Rhabdomyosarcoma (RMS) is a highly malignant tumor that is the most common form of soft tissue tumors in children. It is thought to arise as a consequence of myogenic precursors failing to differentiate into normal muscle [1]. There are two major histological categories of RMS, the embryonal (ERMS) and alveolar (ARMS) subtypes. The more common form of the disease is the ERMS subtype, characterized by loss of heterozygosity at the 11p15 locus, a region which harbors insulin-like growth factor 2 (IGF2). ARMS, the more aggressive form of RMS, is characterized by t(2;13)(q35;q14) or t(1;13)(q36;q14) translocations in many of the tumors which result in chimeric transcripts that fuse the 5′ DNA binding domain of PAX3 or PAX7, respectively, to the transactivation domain of a forkhead transcription factor, creating novel PAX3/7-FOXO1 fusion proteins [2,3].

Normal myogenesis is controlled by the concerted activity of the myogenic regulatory factors (MRF), a group of four highly related bHLH transcription factors composed of Myf5, MyoD, Myf6, and myogenin [4]. Myf5 and MyoD function early in the commitment steps of myogenesis [5]. Myf6, also known as MRF4, is thought to act both early in myogenesis and later in both myotube formation and adult muscle maintenance [6]. Myogenin is involved in the later stages of differentiation by promoting efficient myoblast fusion and the differentiation of mature skeletal muscle fibers [7,8].

The MRFs form avid heterodimers with E-proteins *in vitro*, and are thought to function as heterodimers *in vivo*[9]. Both the E2A splice variants, E12 and E47, and HEB appear to function in myogenesis [9,10]. Recent work has shown that E protein interactions can mediate differentiation in RD cells, which were derived from an ERMS tumor [11]. The myocyte enhancer factor 2 (MEF2) is a regulator of many developmental programs, including myogenesis [12]. MEF2 is encoded by four vertebrate genes which encode MEF2A, MEF2B, MEF2C and MEF2D. The MEF2 family is expressed in distinct but overlapping temporal and spatial expression patterns in the embryo and adult [13]. Both MEF2C and MEF2D are implicated in myogenesis [14,15]. MEF2 factors alone do not possess myogenic activity, but work in combination with the MRFs to drive the myogenic differentiation program [16].

MEF2 proteins control differentiation, proliferation, survival and apoptosis in a wide range of cell types. The N-terminus of the MEF2 proteins contains a highly conserved MADS box and an immediately adjacent motif termed MEF2 domain. Together, these motifs mediate dimerization, DNA binding and co-factor interactions [17]. The C-terminus of the MEF2 proteins is highly divergent among the family members and functions as the transcriptional activation domain. MEF2 proteins function as endpoints for multiple signaling pathways and confer a signal-responsiveness to downstream target genes. MAP kinase pathways are known to converge on MEF2 [18,19], resulting in a phosphorylation of the transcriptional activation domain of MEF2 which augments its transcriptional activity. Calcium signaling pathways also modulate MEF2 activity through multiple mechanisms [20-23]. The activity of MEF2 is tightly controlled by class II HDACs, which bind to the MADS domain and promote the formation of multiprotein repressive complexes on MEF2 dependent genes [24]. Phosphorylation of class II HDACs is mediated by calcium regulated protein kinases, which promote the nuclear-cytoplasmic shuttling of the HDACs and subsequent activation of MEF2C [24,25]. MEF2D promotes late muscle differentiation through use of alternative MEF2D isoforms which generates a muscle specific MEF2Dα2 isoform [26], which binds to the co-activator ASH2L and is resistant to phosphorylation by PKA and association with HDACs [27].

Rhabdomyosarcoma tumors express the myogenic regulatory factors, but the MRFs are unable to promote differentiation [28-30]. Indeed, MyoD and myogenin are used as diagnostic markers for RMS as they are expressed in almost every RMS tumor including both major histological subtypes, embryonal RMS (ERMS) and alveolar RMS (ARMS) [31]. Several cell lines have been derived from RMS tumors and the cell lines exhibit many of the characteristics of RMS tumors. These lines include RD (ERMS), RD2 (ERMS), RH28 (ARMS) and RH30 (ARMS) cell lines. The RMS cell lines express Myf5, MyoD and myogenin, but the proteins appear non-functional [30]. When MRF responsive reporters are transfected into RD cells, little activity is detected [28,29]. Ectopic expression of the MRFs does not rescue the block to differentiation [30], although expression of myogenic co-factors such as E proteins, in conjunction with MyoD, or MEF2C can promote differentiation [11,32].

We have shown here that MEF2D expression is affected at the level of both RNA and protein in four independent RMS cell lines representing both common subtypes of RMS and in primary tumor cells from a mouse model of ERMS. Transfection of MEF2D reactivates muscle specific reporter gene constructs and muscle specific gene expression in both RD (ERMS) and RH30 (ARMS) cell lines. Expression of exogenous MEF2D promotes differentiation as assayed by myosin heavy chain staining in the RH30 ARMS cell line. Consistent with these results, we find that restoration of MEF2D in RH30 cells reduces proliferation, motility and anchorage independent growth *in vitro*. Moreover, the RH30 cells expressing exogenous MEF2D cannot produce tumors in a xenograft model, unlike RH30 cells expressing a vector control.

## Results

### MEF2D is down regulated in RMS cells

**Figure 1:**
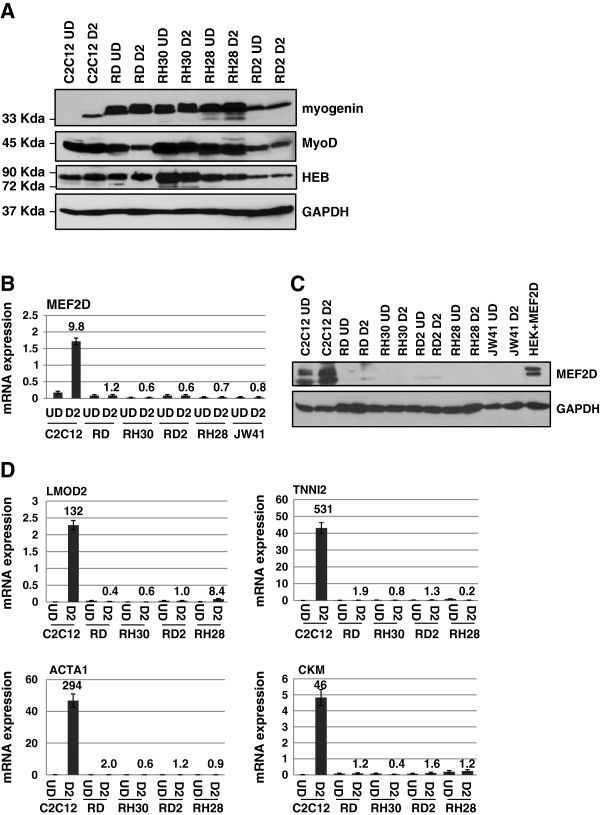
**MRFs and E proteins are expressed in RMS cells, but MEF2D is severely down regulated at the protein and RNA level. A**. RMS cell lines express myogenin, MyoD and HEB. Extracts from each indicated cell line were western blotted and probed with antibodies against myogenin, MyoD, HEB and GAPDH. UD represents undifferentiated (proliferating) cells and D2 represents cells subjected to differentiation conditions for 2 days. **B**. *MEF2D* gene expression levels are down regulated in RMS cells. Gene expression was assayed for *MEF2D* from cell lines indicated as in **A**. **C**. MEF2D protein expression is down regulated in RMS cells. Protein extracts from cell lines indicated as in **A**. were used for western blots and probed with antibodies against MEF2D or GAPDH. **D**. Muscle specific genes are highly down regulated in RMS cells. mRNA expression for the indicated genes is shown for the indicated cell lines while proliferating (UD) and after differentiation for two days (D2). The number above the bars in the graphs represent the fold change between the UD and D2 samples.

To understand the deregulation of myogenesis in RMS cells, we first determined the level of myogenin, MyoD and associated co-factors in RMS cells in comparison to the normal expression levels present during skeletal muscle differentiation (Figure 1A). Four independently derived RMS cell lines were used for this analysis. The ERMS subtype was represented by RD and RD2 cells and the ARMS subtype was represented by RH30 and RH28 cells. Murine C2C12 cells, a commonly used myogenic cell line, were used as a comparative cell line for RMS cells. Myogenin was not detectable in proliferating myoblasts, but was strongly induced upon differentiation. MyoD was expressed in proliferating myoblasts and maintained expression during differentiation. We found that myogenin was expressed in all assayed RMS cell lines (Figure 1A). The levels of myogenin in most RMS lines were higher than the level observed in normal differentiating myoblasts. The level of myogenin observed in RD2 cells was not as robust as was observed in the other RMS lines, but the level was still similar or modestly higher than that observed in normal differentiating myoblasts. We also assayed for MyoD expression and found that the expression of MyoD was similar to the expression of MyoD observed in myoblasts (Figure 1A). The cell lines of the ARMS subtype, RH30 and RH28, expressed MyoD at levels comparable or slightly higher to that observed in normal myoblasts. While expressed at a lower level than that found in ARMS cells, MyoD expression was also detected in both cell lines of the ERMS subtype, RD and RD2.

Next, we assayed the expression profile of the co-factors required by myogenin in C2C12 and RMS cells. We looked for the E proteins by assaying for both the E2A variants and HEB. The E2A locus encodes the two slice variants, E12 and E47, which differ by differential use of a single exon [33]. E12/47 and HEB are known to be expressed in proliferating and differentiating myoblasts. We found that the RMS cell lines showed apparently normal levels of expression of HEB (Figure 1A). RD and RH30 cell lines were used to confirm expression of E12/47 and we again observed high levels of the E proteins (Additional file 1: Figure S1).

We next examined the expression of the MEF2 family in C2C12 cells and RMS cells and found that while MEF2A, MEF2B and MEF2C were expressed (Additional file 1: Figure S2), MEF2D was dramatically down regulated in RMS cells when compared to the levels found in C2C12 cells (Figure 1B). The down regulation of MEF2D was also observed in primary cells derived from a mouse model of ERMS, JW41 (Figure 1B). The expression of MEF2D at the protein level was determined from extracts from proliferating cells and cells that were induced to differentiate for two days. MEF2D was robustly expressed in C2C12 cells, but was greatly reduced in all RMS cell lines tested (Figure 1C). HEK293 cells expressing exogenous MEF2D were used to confirm specificity of the antibody. Extracts from HEK293 cells expressing MEF2D were not recognized by antibodies against MEF2C and extracts from HEK293 cells expressing MEF2C were not recognized by antibodies against MEF2D (Additional file 1: Figure S3).

To confirm that muscle specific genes were down regulated in RMS cells, we assayed for the expression of several differentiation specific genes in C2C12 cells and RMS cell lines. Genes chosen for analysis were leiomodin2 (*LMOD2*), troponin I type 2, skeletal, fast (*TNNI2*), creatine kinase, muscle (*CKM*) and actin (*ACTA1*). We found that, as anticipated, these genes were robustly up regulated in response to differentiation in C2C12 cells. However, expression of these genes was at baseline levels in RMS cells and expression was not significantly induced by exposure to differentiation conditions (Figure 1D).

### MEF2 is not associated with muscle specific promoters while MRFs and E proteins are present

**Figure 2:**
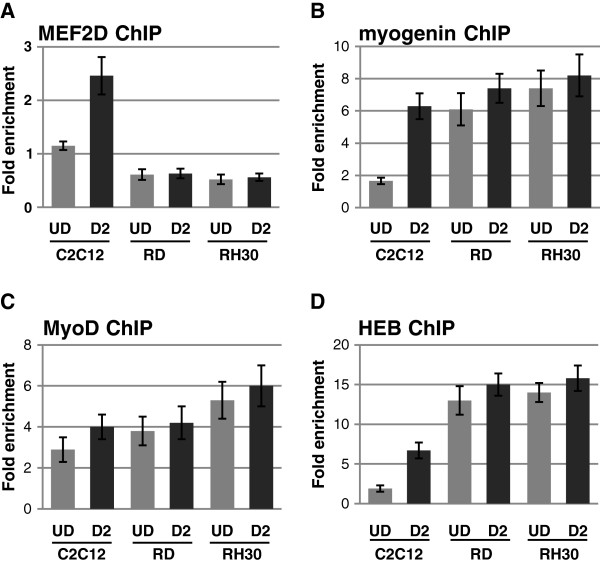
**Myogenin, MyoD and HEB associate with the *TNNI2 *promoter while MEF2D does not.** ChIP assays in RD and SJRH30 cells were performed with antibodies against MEF2D **(A)**, myogenin **(B)**, MyoD **(C)** and HEB **(D)**. Normal rabbit IgG was used as a negative control. Samples were analyzed with primers corresponding to the *TNNI2* promoter.

To determine if the loss of MEF2D affects promoter occupancy in RMS cells, chromatin immunoprecipitation assays were performed. We first assayed for the presence of MEF2D at muscle specific promoters. While MEF2D was highly down regulated, it was possible that low levels of MEF2D present in RMS cells could be associated with DNA. However, we were unable to detect MEF2D at the promoter of any gene tested. Shown are data from the *TNNI2* promoter (Figure 2A), but the promoters of *LMOD2*, desmin *(DES)* and *CKM* were also assayed with similar results (data not shown). To determine if the MRFs and associated co-factors were present at promoters in the absence of MEF2D, we assayed for the presence of myogenin, MyoD and HEB as we have previously shown that myogenin, MyoD and HEB bind these promoters during normal myogenesis [34]. Here, we found that myogenin (Figure 2B), MyoD (Figure 2C) and HEB (Figure 2D) were bound to muscle specific promoters in RD and RH30 cells. As the MRF and E-protein binding profiles were unaffected by the down regulation of MEF2D, these data suggest that the lack of MEF2D proteins in RMS cells does not affect the binding of the MRFs or associated co-factors to muscle specific promoters, but is likely significant to the inactivity of the MRFs in RMS cells.

### Exogenous expression of MEF2D activates muscle specific reporters

**Figure 3:**
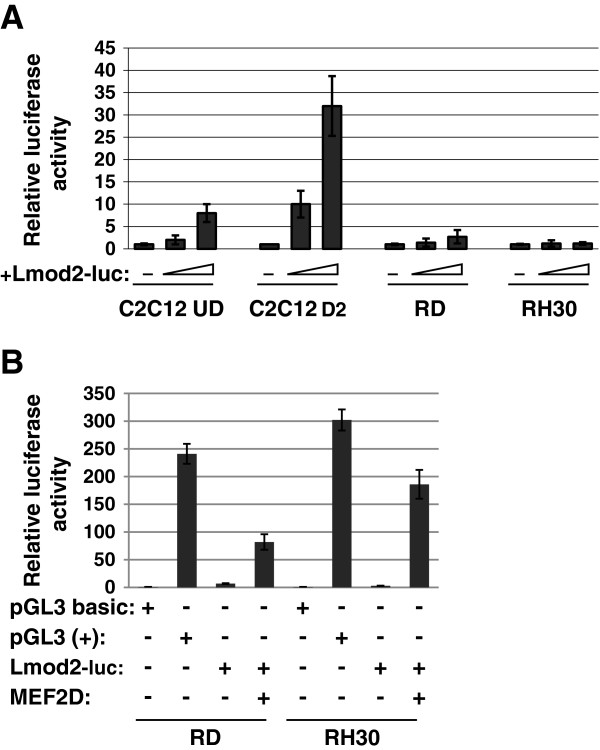
**Muscle specific reporters are largely inactive in RMS cells but can be stimulated by exogenous MEF2D. A**. Muscle specific reporters show minimal activity in RD cells, but are completely inactive in RH30 cells. Indicated cell lines were transiently transfected with increasing concentrations of a muscle specific reporter construct. A luciferase reporter containing the *leiomodin* (*Lmod2*) promoter was used. pGL3 basic was used as the negative control. This value was set to 1, and all other values presented are relative to this value. pGL3+, which contains the constitutive CMV promoter, was used as the positive control. Error bars represent standard deviation from the mean for the three replicate samples for each data point. **B**. Exogenous MEF2D promotes expression of the *Lmod2* reporter construct. MEF2D was transfected into RD and RH30 cells. Assay was performed as described in **A**.

To determine if the loss of MEF2D contributed to the inactivity of muscle specific genes RMS cells, we assayed for activity using muscle specific luciferase reporters. We used several muscle specific reporters that show differentiation specific expression and respond to both myogenin and MyoD [35,36]. Data from all tested reporters were similar and data for the *Lmod2*-luciferase reporter are shown. We have previously characterized the expression of these reporters and shown that they are active in differentiated C2C12 cells, consistent with the expression pattern of myogenin, and inactive in non muscle cells such as NIH3T3 cells [35,36]. The *Lmod2* reporter construct was transfected into RD and RH30 cell lines and assayed for luciferase expression (Figure 3A). In the ERMS line, RD, the *Lmod2* reporter had minimal activity that was modestly above baseline values. The *Lmod2* reporter was completely inactive in the ARMS cell line, RH30. The modest activity of the reporter in RD cells is interesting as it suggests that the degree of block to MRF function correlates with the oncogenic potential of the tumor type.

We next co-transfected MEF2D with the muscle specific reporters and assayed for expression. The muscle specific MEF2Dα2 isoform [26] was chosen for our study. Shown are the results for the *Lmod2* reporter. We found that transfection of MEF2D promoted expression of the *Lmod2* reporter in RD and RH30 cells, with a more robust effect noted in RH30 cells (Figure 3B). Exogenous MyoD and myogenin were also tranfected with or without MEF2D but we found that this did not further stimulate the activation conferred by MEF2D alone (data not shown). As MEF2D requires the MRFs to function [16,37], the data suggest that the endogenous levels of MyoD and myogenin in RD and RH30 cells are sufficient to stimulate the activation driven by MEF2D.

### Expression of MEF2D activates muscle specific gene expression in RMS cells

**Figure 4:**
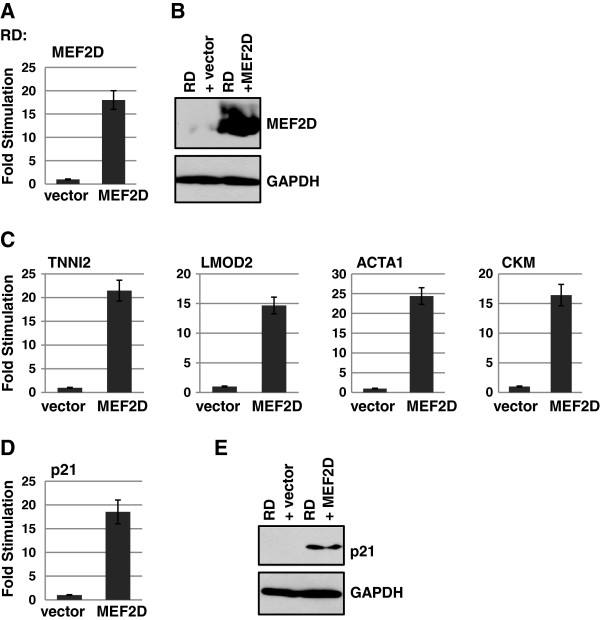
**MEF2D activates muscle specific gene expression in ERMS cells.** RNA expression of MEF2D was determined by qPCR **(A)** and protein expression confirmed by western blot **(B)** following transient tranfection of RD cells with an expression construct for MEF2D or a vector only control. **C**. Muscle specific gene expression is activated by MEF2D. qPCR for the indicated genes is shown. **D**. p21 is activated by MEF2D at the level of RNA and protein **(E)**.

Our data suggested that the loss of MEF2D might be responsible for the failure of RMS cells to differentiate, so we next assayed if exogenous expression of MEF2D could restore muscle specific gene expression and promote differentiation in RMS cells. RD and RH30 cells were transfected with a vector only control and an expression construct for MEF2D and stable drug resistant clones were selected. However, stable cell lines overexpressing MEF2D were not recovered for RD cells despite multiple experimental attempts. TUNEL analysis revealed a high level of apoptosis in the transfected cells (data not shown). Thus, we transiently transfected RD cells with vector control or MEF2D and examined the effect on muscle specific genes. We also assayed for the expression of the cyclin-dependent kinase (Cdk) inhibitor p21^CIP1/WAF1^ (*CDKN1A*) which is induced early in myoblast differentiation and functions to block cell cycle progression [38,39]. Induction of p21 in RMS cells is correlated with growth arrest and differentiation of RMS cells [40-42] and is required for ceramide-induced G2 arrest [43]. We confirmed the expression of exogenous MEF2D in RD cells at the RNA (Figure 4A) and protein level (Figure 4B). We found that MEF2D expression led to an upregulation of muscle specific genes (Figure 4C) and the differentiation specific gene *CDKN1A* (p21) at the level of RNA (Figure 4D) and protein (Figure 4E).

**Figure 5:**
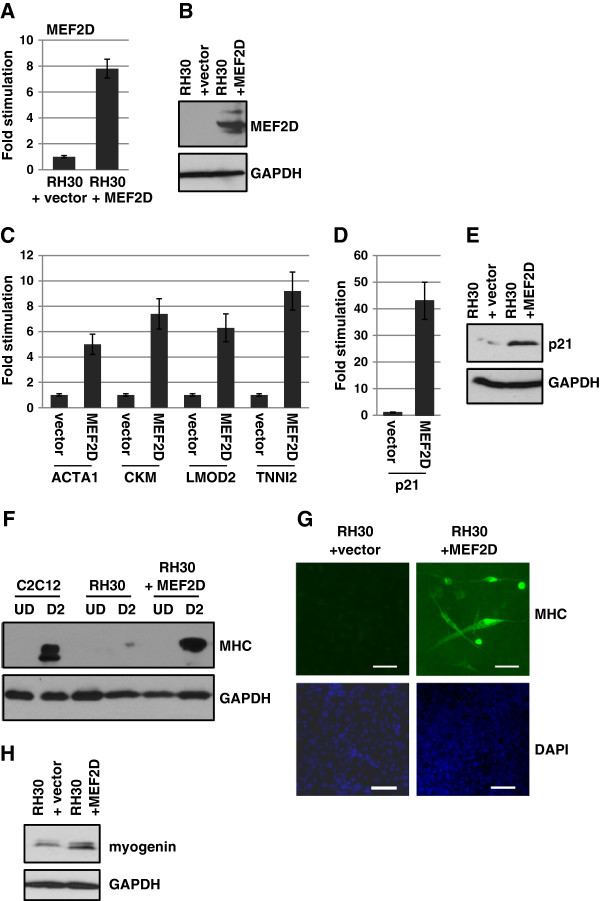
**MEF2D promotes muscle differentiation in ARMS cells. A**. The expression of MEF2D was confirmed by RNA and western blot **(B)** analysis in RH30 cells stably transfected with an expression construct for MEF2D or a vector only control. **C**. MEF2D expression activates muscle specific genes. Quantitative gene expression analysis of the indicated endogenous muscle specific genes in RH30 cells stably transfected with MEF2D or vector only expression constructs. **D**. MEF2D expression activates p21 (*CDKN1A*) expression at the level of RNA and protein **(E)**. **F**. MEF2D promotes myosin heavy chain expression. Western blots were probed with antibodies against myosin heavy chain (MyHC) and GAPDH. **G**. MEF2D promotes myofiber formation. RH30 cells transfected with MEF2D or vector control were immunostained with myosin heavy chain antibodies. DAPI staining is also shown. Fluorescent microscopy images were taken at 200X and scale bars represent 10 μms. **H**. MEF2D expression upregulates myogenin. Western blot was probed with antibodies against myogenin and GAPDH.

Stable RH30 cell lines overexpressing MEF2D were recovered and screened to confirm expression at the level of RNA (Figure 5A) and protein (Figure 5B). RH30 cells transfected with vector only control or MEF2D were induced to differentiate for 2 days and gene expression analysis revealed an induction of differentiation specific gene expression in the presence of MEF2D at each gene tested (Figure 5C). We also found that expression of *CDKN1A* (p21) was robustly stimulated upon differentiation in the presence of MEF2D at the level of RNA (Figure 5D) and protein (Figure 5E). We also examined myosin heavy chain (MHC) expression, a hallmark of differentiated cells. As anticipated, C2C12 cells expressed low levels of MHC while proliferating, but MHC expression was strongly induced in differentiated cells (Figure 5F). In RH30 cells, almost no induction of MHC could be detected upon differentiation. However, RH30 cells tranfected with MEF2D robustly restored MHC expression upon differentiation (Figure 5F). RH30 cells transfected with MEF2D or vector controls were also immunostained with myosin heavy chain antibodies following exposure to differentiation conditions for 2 days. While myosin heavy chain positive cells could not be identified in RH30 cells transfected with a vector control, myosin heavy chain positive cells, including multinucleated myofibers, were readily observed in RH30 cells expressing MEF2D (Figure 5G). We also assayed for up regulation of myogenin as a marker of differentiation and found that myogenin was up regulated in the presence of MEF2D upon differentiation (Figure 5H). Thus, these results are highly suggestive that the lack of MEF2D is implicated in the failure of RMS cells to differentiate.

### MEF2D inhibits the proliferation, migration and anchorage independent growth of SJRH30 cells *in vitro* and inhibits RMS tumor growth *in vivo*

**Figure 6:**
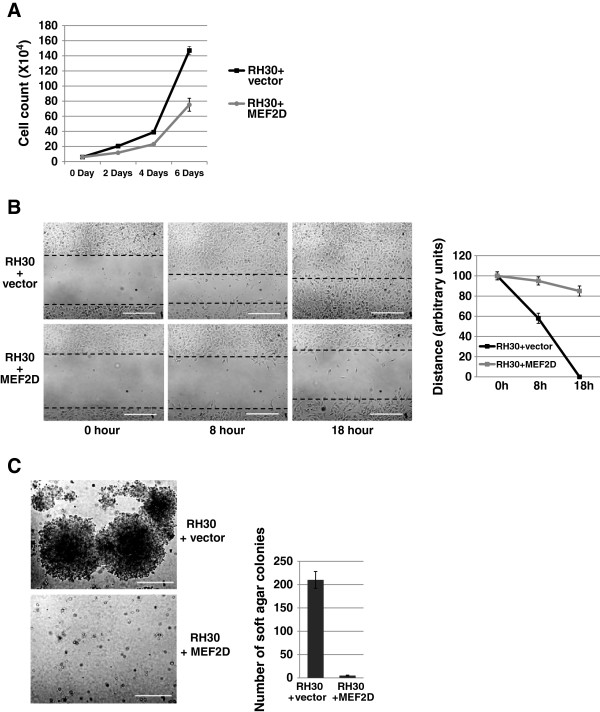
**Expression of MEF2D inhibits proliferation, migration and anchorage independent growth of SJRH30 cells. A**. MEF2D inhibits the proliferation of RH30 cells. Cells were seeded at equivalent densities and harvested for cell counts every two days. Error bars, SD. **B**. MEF2D inhibits the migration of RH30 cells. Monolayer of confluent cultures were lightly scratched with a pipette tip and phase contrast images were taken immediately after wounding (0 h) or at indicated times. Left panel: images were taken at 100X magnification and scale bars represent 10 μms. Right panel: data were quantified with a statistical evaluation of the distance between the borders (dotted lines, left panel) in three independent assays. Error bars, SD. **C**. MEF2D inhibits anchorage independent growth of RH30 cells. Soft agar colony formation was assayed in RH30 cells expressing MEF2D or vector control. Left panel: images are as described in **B**. Right panel: three independent assays were quantified. Error bars, SD.

To evaluate the effect of MEF2D expression on cell proliferation, we measured the growth rate of RH30 cells with vector control or with MEF2D. We found that the expression of MEF2D inhibited the proliferation rate of RH30 cells by approximately 2 fold (Figure 6A). To assay for cell migration, we used the scratch wound assay. After 8 hours the wounds were colonized to a much higher degree by RH30 cells with vector control than RH30 cells with MEF2D (Figure 6B). This difference was still obvious at 18 hours after wounding. The degree to which wound healing was delayed appears to be beyond what could be attributed to the modest growth defect observed in the cells. Next, we examined the effects of MEF2D expression on attachment independent clonal growth of cells in a soft agar assay, a hallmark of cell transformation. We found that RH30 cells showed a strong capacity for colony formation in this assay and that MEF2D expression almost entirely blocked the ability of RH30 cells to grow in an anchorage independent manner (Figure 6C). The modest growth delay in MEF2D expressing cells cannot account for the lack of clonal growth observed in this assay as cells were grown for 30 days in soft agar.

**Figure 7:**
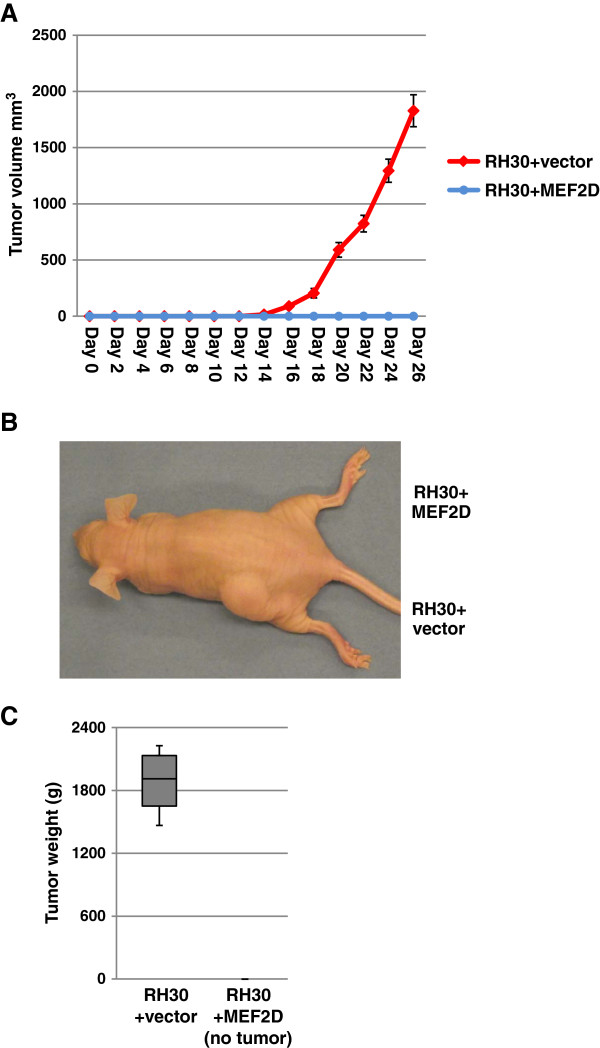
**Expression of MEF2D inhibits tumor formation *in vivo*. A**. Time course analysis of tumor growth after s.c. injection of 2 × 10^6^ RH30-vector or RH30-MEF2D cells. Eight animals were used. **B**. Tumor growth is shown at 4 weeks. Each animal was injected in the left flank with RH30 cells expressing vector and in the right flank with RH30 cells expressing MEF2D. **C**. Tumor weight from RH30 expressing vector cells. Data from all eight animals are represented.

Finally, we tested whether MEF2D expression in ARMS cells could act as an endogenous antitumor factor *in vivo*. 2 × 10^6^ cells from vector control RH30 cells or RH30 cells expressing MEF2D were injected into the hind limb of nude mice and the tumor size was measured every five days. RH30 cells transfected with a vector control formed visible tumors within the first 2 weeks. In contrast, overexpression of MEF2D led to a complete block of tumor growth (Figure 7A and B). Mice were sacrificed at 4 weeks and tumors resulting from the vector control RH30 cells were dissected, measured and weighed. The overall tumor sizes in each case were comparable (Figure 7C).

## Discussion

Here, we have shown that MEF2D is highly down regulated in four independently derived RMS cell lines representing the two major subtypes of RMS as well as primary cells derived from an ERMS model of RMS. Reestablishment of MEF2D expression in both RD cells, which represent the ERMS subtype and RH30 cells, which represents the ARMS subtype, activates muscle specific gene expression and the cell cycle regulator p21, suggesting that the loss of MEF2D contributes to the inactivity of myogenin and MyoD in RMS cells and inhibits differentiation. Our results suggest that the down regulation of *MEF2D* is a common feature in both common subtypes of RMS. Significantly, we have found that restoring MEF2D expression in these cells impairs the ability of RH30 cells to migrate and grow in an anchorage independent manner *in vitro* and form tumors *in vivo.* Thus, MEF2D appears to significantly prevent the oncogenic growth properties of the aggressive ARMS subtype of RMS.

The regulation of *MEF2D* is not currently understood, but the lack of expression in both subtypes of RMS suggests that a common pathway contributes to the silencing, such as the inactivity of the MRFs. The MRFs may promote the expression of MEF2D which is then required for MRF activity on differentiation specific genes. MEF2D cooperates with MyoD to recruit RNAPII and activate transcription at late gene promoters [15]. Myogenin cooperates with MEF2D to recruit the Brg1 ATP-dependent chromatin remodeling enzyme to alter chromatin structure and promote late muscle gene expression [37]. Understanding the regulation of MEF2D will be an important future direction for our studies in efforts to understand how to reactivate this critical regulator of cell growth and differentiation in RMS cells.

Alterations in the activity or expression of the MEF2 family have previously been implicated in RMS. Inactivation of the p38 MAP kinase has been shown to contribute to RMS and the enforced expression of an activated MAP kinase restored MyoD function and enhanced MEF2 activity in a GAL4 tethered reporter assay [44]. In this work, it was suggested that the enhancement of MEF2 activity by p38 could contribute to the rescue of myogenic program in RMS cells [44]. It has also been shown that MEF2 dependent reporters have reduced activity in RMS cells and that the reduced activity of GAL4-MEF2 can be induced by expression of the steroid receptor co-activator SRC-2 [45]. A previous study which assayed gene expression changes in a murine model of alveolar rhabdomyosarcoma detected a down regulation of *Mef2c* in these tumors [46]. It has also been shown that expression of MEF2C in RD cells promotes the expression of differentiation specific genes [32]. Taken together, the data suggest that the entire MEF2 family may be inactivated through multiple mechanisms in RMS cells and fully understanding the inactivation of the MEF2 family will be essential in understanding the pathology of RMS cells.

The activity of MEF2 proteins is influenced by variety of intracellular signaling pathways and by interaction with many coactivators and corepressors. Class II histone deacetylases (HDAC), which include HDAC-4, -5,-7 and −9, are central regulators of MEF2C activity [24,47-49]. Class II HDACs inhibit MEF2 activity and it has been shown that MEF2 regulates HDAC9 gene expression in a negative feed forward regulatory loop [50]. MEF2D employs alternative isoforms to regulate differentiation. The ubiquitously expressed MEF2Dα1 is phosphorylated by PKA and bound by HDACs to function as a transcriptional repressor, while the muscle specific MEF2Dα2 isoform is resistant to phosphorylation and binds to the co-activator ASH2L [27]. An important future area of study will be the deregulation of HDACs and potentially the isoform usage of the MEF2 proteins that may occur in RMS cells and account for the inactivity of the MEF2 family.

A surprising aspect of this study was the dramatic effect of MEF2D on cell motility, migration, anchorage independent growth and tumor growth *in vivo*. This suggests that MEF2D plays an important role in controlling the gene expression of factors that control this important process. It is surprising that the restoration of a single transcriptional co-activator could have such a large effect on the oncogenic properties of these cells. Our results are highly suggestive that restoring MEF2D in RMS cells may effectively impede tumor growth and dissemination.

Our work contributes to the growing body of work that shows that expression of myogenic co-factors can rescue the block to differentiation in RMS cells [11,32] and indicates that deregulation of required co-factors for appropriate muscle specific gene expression is a common mechanism utilized by RMS cells to overcome terminal differentiation signals.

## Conclusions

We have found that MEF2D is silenced in RMS cells representing both common subtypes of the disease. Our work suggests that reactivating MEF2D in RMS cells is an attractive therapeutic target for inhibiting the tumor growth of these cells which may provide new insight into treatment of this pediatric cancer.

## Methods

### Cell culture

RD and SJRH30 (RH30) cells (ATCC) were grown in Dulbecco’s modified Eagle medium (DMEM) supplemented with 10% fetal bovine serum (Hyclone) according to standard protocols. RD2 and RH28 were obtained from Denis Guttridge, Ohio State University, and grown as described above. All cell lines were authenticated by Bio-Synthesis (Lewisville, TX) using STR analysis on September 14, 2011. JW41 cells, isolated from an ERMS tumor from a *p53*^−/−^*/c-fos*^−/−^ mouse [51], were the gift of Charlotte Peterson, University of Kentucky. Proliferating C2C12 myoblasts (ATCC) and HEK293 cells (ATCC) were grown in DMEM supplemented with 10% fetal bovine serum (Hyclone). To induce differentiation of C2C12 myoblasts into myotubes, cells were grown to 70% confluence and the media switched to DMEM supplemented with 2% horse serum (Hyclone). C2C12 cells were grown in differentiation medium for the number of days indicated in each experiment.

### Western blot analysis

Cell extracts were made by lysing PBS washed cell pellets in radio-immunoprecipitation assay buffer (RIPA) supplemented with protease inhibitors (Complete protease inhibitor, Roche Diagnostics). Following incubation on ice, clear lysates were obtained by centrifugation. Protein concentrations were determined by Bradford’s assay (Bio-Rad). For each sample, 30 μg of protein was loaded on each gel. Proteins were transferred onto a PVDF membrane using a tank blotter (Bio-Rad). The membranes were then blocked with 5% milk and 1X Tris buffered saline plus tween 20 (TBST) and incubated with primary antibody overnight at 4°C. Membranes were then washed with 1X TBST and incubated with the corresponding secondary antibody. Membranes were again washed with 1X TBST, incubated with chemiluminescent substrate according to manufacturer’s protocol (SuperSignal, Pierce) and visualized by autoradiography. The antibodies used include anti-MEF2D (P-17, Santa Cruz Biotechnologies), anti-MEF2C (E-17, Santa Cruz Biotechnologies), anti-HEB (A-20, Santa Cruz Biotechnologies), anti-myogenin (F5D, Developmental Studies Hybridoma Bank), anti-MyoD (5.8A, Santa Cruz Biotechnologies), anti-MHC (MF-20, Developmental Studies Hybridoma Bank) and anti-GAPDH (Millipore).

### Gene expression analysis

RNA was isolated from cells by Trizol extractions (Invitrogen). Following treatment with DNase (Promega), two micrograms of total RNA was reversed transcribed with MultiScribe™ MuLV reverse transcriptase (Applied Biosystems). cDNA equivalent to 40 ng was used for quantitative polymerase chain reaction (qPCR) amplification (Applied Biosystems) with SYBR green PCR master mix (Applied Biosystems). Samples in which no reverse transcriptase was added (no RT) were included for each RNA sample. The relative levels of expression of genes were normalized according to those of hypoxanthine guanine phosphoribosyl transferase (*HPRT*). qPCR data were calculated using the comparative Ct method (Applied Biosystems). Standard deviations from the mean of the [Δ] Ct values were calculated from three independent RNA samples. Primers are described in Additional file 1: Table S1. Where possible, intron spanning primers were used. All quantitative PCR was performed in triplicate and three independent RNA samples were assayed for each time point. qPCR gene expression data are shown using two formats. For measurements of relative gene expression (fold stimulation), a fold change was calculated for each sample pair and then normalized to the fold change observed at *HPRT*. For relative measurements of mRNA expression levels (mRNA expression), gene expression levels were quantitated using a calibration curve based on known dilutions of concentrated cDNA. Each mRNA value was normalized to that of *HPRT*. Fold change was calculated by dividing the mRNA expression values of each sample pair.

### Chromatin immunoprecipitation

ChIP assays were performed and quantified as described previously [34] with the following modifications: 1 × 10^7^ cells were used for each immunoprecipitation and protein A agarose beads (Invitrogen) were used to immunoprecipitate the antibody:antigen complexes. The following antibodies were used: anti-MEF2D (P-17, Santa Cruz Biotechnology), anti-MyoD (5.8A, Santa Cruz Biotechnology), anti-myogenin (F5D, Developmental Studies Hybridoma Bank), anti-HEB (A-20, Santa Cruz Biotechnology). Rabbit IgG (Santa Cruz Biotechnology) was used as a non-specific control. Primers are described in Additional file 1: Table S1. The real time PCR was performed in triplicate. Values of [Δ] [Δ] Ct were calculated using the following formula based on the comparative Ct method: Ct, template (antibody) - Ct, template (IgG) = [Δ] Ct. Fold enrichments were determined using the formula : 2 ^- [Δ] Ct.^ (experimental)/2 ^-[Δ]^^Ct^ (reference, CHR19). Standard error from the mean was calculated from replicate [Δ][Δ] Ct values obtained from at least three individual experiments.

### Cell transfections and luciferase assays

RD or RH30 cells were transfected with calcium phosphate according to standard protocols. The plasmids EMSV-myogenin (gift of D. Edmondson, U.T. Medical School at Houston) and pEMCIIs (provided by Andrew Lassar, Harvard Medical School) were used for expressing myogenin and MyoD, respectively. The plasmids pcDNA-MEF2C and pcDNA-MEF2D (gift of Eric N. Olson, University of Texas Southwestern Medical Center) were used for expressing MEF2C and MEF2D, respectively. pcDNA-MEF2D contains the MEF2Dα2 isoform of MEF2D. Luciferase activity was determined using the Dual-Luciferase Reporter Assay System (Promega). RH30 or RD cells were seeded at a density of 5 × 10^3^ cell per well in 96 well plates and transfected with 0.4 ug of DNA. Transfections were normalized to Renilla luciferase. Transfections were performed in triplicate and all data sets were repeated at least twice.

### Stable cell lines

Stable SJRH30 cell lines overexpressing exogenous MEF2D were made by transfecting SJRH30 cells with linearized pcDNA-MEF2D plasmid or the empty vector, linearized pcDNA3.1, and selecting for geneticin (400 ug/ml) resistant colonies. Individual clones were isolated and propagated.

### Immunohistochemistry

Cells were grown on cover slips, fixed with paraformaldehyde, incubated with goat serum and 1.0% NP-40 for one hour and washed with PBS. Primary antibodies against myosin heavy chain (1:100, MF20, Developmental Studies Hybridoma Bank) were incubated overnight at 4°C, washed with PBS and detected by Alexa Fluor-488 goat anti-mouse antibody (1:500, Invitrogen). Cell nuclei were then stained by incubating with DAPI (1 μM, Invitrogen) for 5 min.

### Proliferation

Cells were seeded in a six well plate at 6 × 10^4^ per well and harvested every two days for cell counts with a hemocytometer. All counts were performed in triplicate and individual experiments repeated three times.

### Scratch wound assay

Cells were grown to 100% confluency and the cell monolayer was scraped in a straight line to create a “scratch” with a p200 pipet tip. The debris was removed and the edge of the scratch smoothed by washing the cells once with 1 ml of growth medium. Markings were created near the scratch to obtain the same field during the image acquisition. The tissue culture dish was then placed in a tissue culture incubator at 37°C for 0–18 hours.

### Soft agar assay

Soft agar assays were carried out in 60 mm dishes in which 2 ml of 0.7% Noble agar (USB) in 1X DMEM with 10% FBS was overlaid with 2 ml of 0.35% agar in 1X DMEM with 10% FBS containing the cells. RH30-pcDNA3.1 (vector) and RH30-MEF2D cells were grown to 100% confluence, trypsinized, and dispersed. Cells of each clone (3 × 10^5^) were plated in triplicate. 1 ml of culture medium was added to the top of each plate every 5 days and cells were grown at 37°C for 30 days. The plates were stained with 1 ml of 0.05% Crystal Violet (Fisher) for > 1 hour and colonies were counted using a dissecting microscope.

### Xenograft

For *in vivo* tumor formation, cells were harvested by trypsin treatment and counted. Cells were washed with PBS and suspended at 10^6^ cells/100 μl in PBS. 2 × 10^6^ cells were subcutaneously injected into the hind flanks of 10 week old female athymic nude mice (*Foxn1*^*nu*^*/Foxn1*^*nu*^*,* Jackson Laboratory). Eight animals were used, and each animal was injected with RH30-pcDNA3.1 cells in the right flank and RH30-MEF2D cells in the left flank. Mice were monitored every other day and tumor dimensions were measured with electronic calipers. Tumor size was estimated by using the modified ellipsoid formula 1/2(length × width^2^). All animal experiments were conducted according to procedures approved by the Institutional Animal Care and Use Committee at Southern Illinois University.

### Statistics

qPCR data are presented as means ± standard deviation (SD). Tumor volume data are also presented as means ± standard deviation (SD). Tumor weight data are represented with a box plot, a graphical description of groups of numerical data through quartiles. Statistical comparisons were performed using unpaired two-tailed Student’s *t* tests, with a probability value of <0.05 taken to indicate significance.

## Competing interests

The authors declare that that they have no competing interests.

## Authors’ contributions

MZ performed all the described experiments. JT made the initial observation that the MEF2 family was deregulated in RMS. MZ and JD analyzed the data and wrote the manuscript. All authors read and approved the final manuscript.

## Supplementary Material

Additional file 1: Figure S1 E proteins are expressed normally in RMS cells. Extracts from the indicated cell lines were normalized for total protein concentration by Bradford assays and used for western blot analysis. The blots were probed with antibodies against E2A (V-18, SCBT) and GADPH (6C5, Millipore). **Figure S2**. MEF2A, MEF2B and MEF2C are expressed in RMS cells. A. Quantitative gene expression analysis was performed on cDNA derived from each of the indicated cell lines. Real time PCR was performed in triplicate on three independent RNA isolations. Data is plotted as mRNA expression levels and error bars indicate standard deviation from the mean. B. Western blot data for MEF2A, MEF2B and MEF2C. HEK293 cells transfected with individual MEF2 expression constructs were used as positive controls. Antibodies used included anti-MEF2A (#9736,Cell Signaling), anti-MEF2B (ab33540, Abcam) and anti-MEF2C (E-17, SCBT). Protein extracts were normalized prior to loading. **Figure S3**. Characterization of antibodies against MEF2. A. An antibody against MEF2C recognizes MEF2C and does not cross react with MEF2D. HEK cells transiently transfected with plasmids encoding MEF2C, MEF2D or the empty vector (pcDNA) were harvested for protein and used for western blot analysis. Blot was probed with anti-MEF2C antibodies (E-17, Santa Cruz Biotechnologies). B. An antibody against MEF2D recognizes MEF2D and does not cross react with MEF2C. HEK cells transiently transfected as in A. were used for western blot analysis. Blot was probed with anti-MEF2D antibody (P-17, Santa Cruz Biotechnologies). **Table S1**. Primers used in study. Click here for file
