# Supplementary material for: Loss of MEF2D expression inhibits differentiation and contributes to oncogenesis in rhabdomyosarcoma cells
Source: Mol Cancer. 2013 Nov 27;12:150. doi: 10.1186/1476-4598-12-150 (PMC3879063; doi:10.1186/1476-4598-12-150)
Supplement: Additional file 1: Figure S1 — E proteins are expressed normally in RMS cells. Extracts from the indicated cell lines were normalized for total protein concentration by Bradford assays and used for western blot analysis. The blots were probed with antibodies against E2A (V-18, SCBT) and GADPH (6C5, Millipore). Figure S2. MEF2A, MEF2B and MEF2C are expressed in RMS cells. A. Quantitative gene expression analysis was performed on cDNA derived from each of the indicated cell lines. Real time PCR was performed in triplicate on three independent RNA isolations. Data is plotted as mRNA expression levels and error bars indicate standard deviation from the mean. B. Western blot data for MEF2A, MEF2B and MEF2C. HEK293 cells transfected with individual MEF2 expression constructs were used as positive controls. Antibodies used included anti-MEF2A (#9736,Cell Signaling), anti-MEF2B (ab33540, Abcam) and anti-MEF2C (E-17, SCBT). Protein extracts were normalized prior to loading. Figure S3. Characterization of antibodies against MEF2. A. An antibody against MEF2C recognizes MEF2C and does not cross react with MEF2D. HEK cells transiently transfected with plasmids encoding MEF2C, MEF2D or the empty vector (pcDNA) were harvested for protein and used for western blot analysis. Blot was probed with anti-MEF2C antibodies (E-17, Santa Cruz Biotechnologies). B. An antibody against MEF2D recognizes MEF2D and does not cross react with MEF2C. HEK cells transiently transfected as in A. were used for western blot analysis. Blot was probed with anti-MEF2D antibody (P-17, Santa Cruz Biotechnologies). Table S1. Primers used in study. [file 1476-4598-12-150-S1.pdf]

Additional File 1

Supplemental Figures and Table for Zhang, Truscott and Davie

Contents:

Figure S1
Figure S2
Figure S3
Table S1

Figure S1

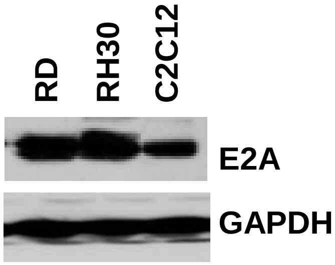

Figure S1. E proteins are expressed normally in RMS cells. Extracts from the indicated cell lines were normalized for total protein concentration by Bradford assays and used for western blot analysis. The blots were probed with antibodies against E2A (V-18, SCBT) and GADPH (6C5, Millipore).

Figure S2

A

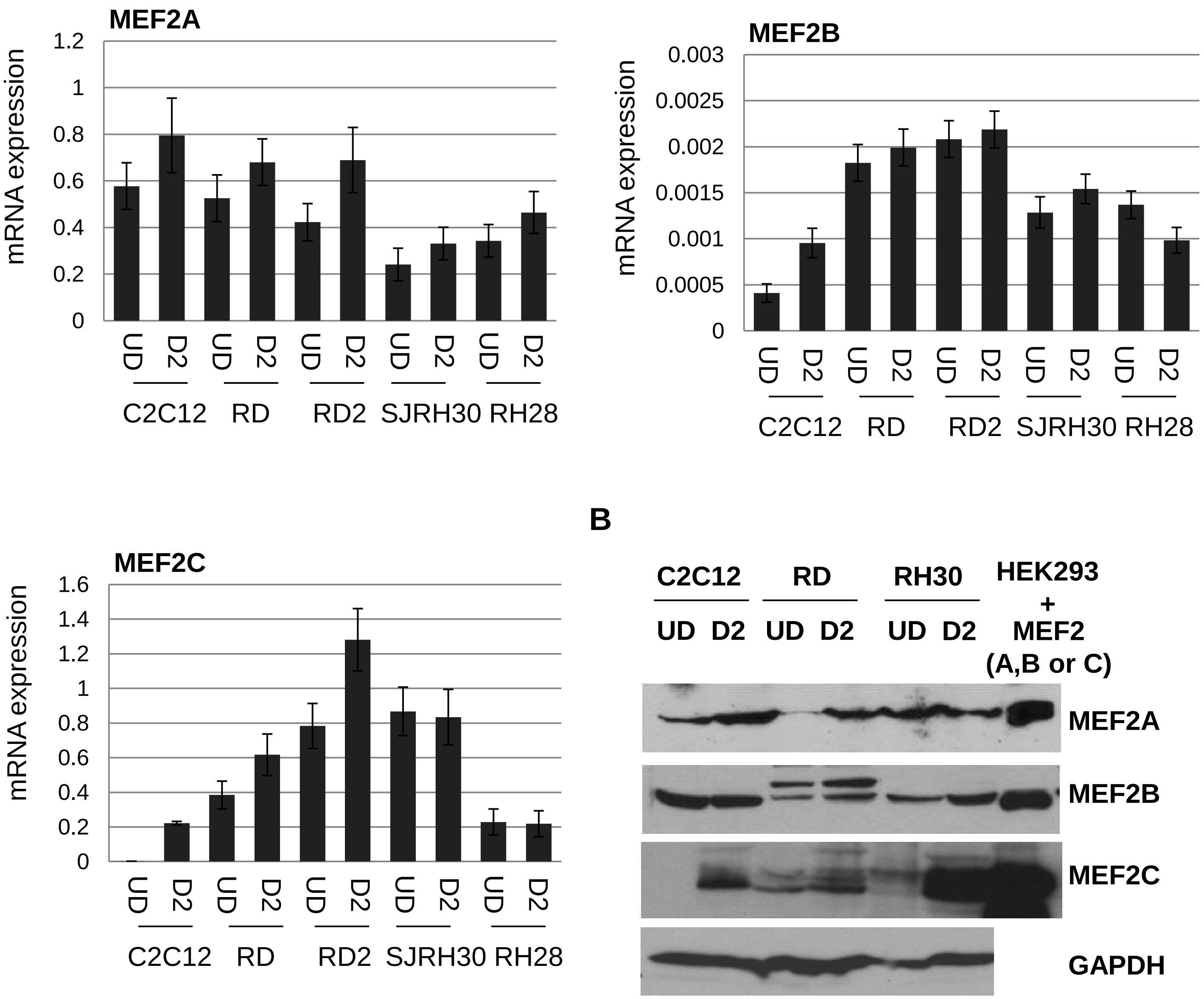

Figure S2. MEF2A, MEF2B and MEF2C are expressed in RMS cells. A. Quantitative gene expression analysis was performed on cDNA derived from each of the indicated cell lines. Real time PCR was performed in triplicate on three independent RNA isolations. Data is plotted as mRNA expression levels and error bars indicate standard deviation from the mean. B. Western blot data for MEF2A, MEF2B and MEF2C. HEK293 cells transfected with individual MEF2 expression constructs were used as positive controls. Antibodies used included anti-MEF2A (#9736,Cell Signaling), anti-MEF2B (ab33540, Abcam) and anti-MEF2C (E-17, SCBT). Protein extracts were normalized prior to loading.

Figure S3

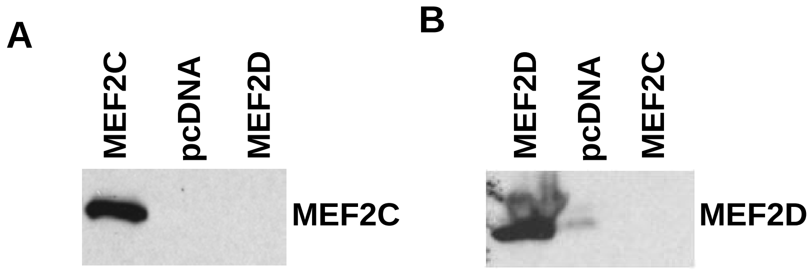

Figure S3. Characterization of antibodies against MEF2. A. An antibody against MEF2C recognizes MEF2C and does not cross react with MEF2D. HEK cells transiently transfected with plasmids encoding MEF2C, MEF2D or the empty vector (pcDNA) were harvested for protein and used for western blot analysis. Blot was probed with anti-MEF2C antibodies (E-17, Santa Cruz Biotechnologies). B. An antibody against MEF2D recognizes MEF2D and does not cross react with MEF2C. HEK cells transiently transfected as in A. were used for western blot analysis. Blot was probed with anti-MEF2D antibody (P-17, Santa Cruz Biotechnologies).

Table S1

Primers used in study

Quantitative gene expression analysis:

human *MEF2D:*	hMEF2D F 5' CTGGTCCCTGTATCTCTCAGC 3'
	hMEF2D R 5' GGTGGGTGGTGACTGTGA 3'
murine *Mef2D*:	mMEF2D F 5' GGGTCATCACTTCCCAGG 3'
	mMEF2D R 5' CCTGGCTGAGTAAACTTGGT 3'
human *LMOD2:*	hLMOD2 F 5' GTGGAAAGGTTGCAGAAGACA 3'
	hLMOD2 R 5' TCGTCACTGTCTTCTTCCTCCT 3'
murine *Lmod2:*	mLMOD2 F 5'ACCTTATCCCGATTTGCTGAAG 3'
	mLMOD2 R 5' ACCTTGAGCATGTCTGCAATG 3'
human *TNNI2:*	hTNNI2 F 5' CAGCACCTGAAGAGTGTGATG 3'
	hTNNI2 R 5' GGTAGTTCTGCTTCTCTGCCTC 3'
murine *Tnni2:*	mTNNI2 F *5'* GCCGCCGAGAATCTGAGA *3'*
	mTNNI2 R *5'* GACATGGAGCCTGGGATGTG 3'
human *ACTA1:*	hACTA F 5' CAGCGGACAGCGCCAAGTGA 3'
	hACTA R 5' TGAGCCTCGTCGCCCACGTA 3'
murine *Acta1:*	mACTA F 5' GGCACCCAGGGCCAGAGTCA 3'
	mACTA R 5' TCATCCCCGGCAAAGCCAGC 3'
human *CKM:*	hCKM F 5' GCCCTCCAGAACTCCTCCCTGG 3'
	hCKM R 5' CCCCGTGGCGATCCGAGATG 3'
murine *Ckm:*	mCKM F 5' CCAGCCAGCCAGGGTCCCAA 3'
	mCKM R 5' ACTCCTCATCGCCGGCCACA 3'
human *CDKN1A:*	hp21 F 5' GGAACTTCGACTTTGTCACC 3'
	hp21 R 5' CAGTGACAGGTCCACATGG 3'
HPRT:	HPRT F 5' TGACACTGGCAAAACAATGCA 3'
	HPRT R 5' GGTCCTTTTCACCAGCAAGCT 3'

ChIP analysis:

human *TNNI2:*	hTNNI2 F 5' GAGGGATTTCTTCCATGCA 3'
	m/hTNN12 R 5' AGGAGAAAGTGTTCCCAAAATGTC 3'
human *CHR19:*	Chr19: F 5' TGGGAAAACTCTCCAGGAC 3'
	Chr19: R 5' CTTTGGTTGCCTGTGCTT 3'
murine *Tnni2*:	mTNNI2 F' GCCAAAGGAGCAAGAGTTAAAAAT
	m/hTNN12 R 5' AGGAGAAAGTGTTCCCAAAATGTC 3'
murine *IgH*:	IgH F 5' GCCGATCAGAACCAGAACACCTGC 3'
	IgH R 5' TGGTGGGGCTGGACAGAGTGTTTC 3'
